# Expected and minimal values of a universal tree balance index

**Published:** 2025-07-11

**Authors:** Veselin Manojlović, Armaan Ahmed, Yannick Viossat, Robert Noble

**Affiliations:** 1 Department of Mathematics, City St George’s, University of London, Northampton Square, London, EC1V 0HB, United Kingdom.; 2 Department of Applied Math & Statistics, Johns Hopkins University, Baltimore, USA.; 3 Ceremade, CNRS, Université Paris-Dauphine, Université PSL, Paris, France.

## Abstract

Although the analysis of rooted tree shape has wide-ranging applications, notions of tree balance have developed independently in different domains. In computer science, a balanced tree is one that enables efficient updating and retrieval of data, whereas in biology tree balance quantifies bias in evolutionary processes. The lack of a precise connection between these concepts has stymied the development of universal indices and general results. We recently introduced a new tree balance index, $J^{1}$, that, unlike prior indices popular among biologists, permits meaningful comparison of trees with arbitrary degree distributions and node sizes. Here we explain how our new index generalizes a concept that underlies the definition of the weight-balanced tree, an important type of self-balancing binary search tree. Our index thus unifies the tree balance concepts of biology and computer science. We provide new analytical results to support applications of this universal index. First, we quantify the accuracy of approximations to the expected values of $J^{1}$ under two important null models: the Yule process and the uniform model. Second, we investigate minimal values of our index. These results help establish $J^{1}$ as a universal, cross-disciplinary index of tree balance that generalizes and supersedes prior approaches.

## 1 Introduction

Broadly speaking, the balance of a rooted tree is the extent to which its terminal nodes (leaves) are evenly distributed among its branches. Indices that quantify tree balance have important applications in systematic biology (Heard 1992; Mooers and Heard 1997; Purvis and Agapow 2002), mathematical oncology (Scott et al. 2020; Noble et al. 2022; Feder and Gao 2024), and other fields (Leventhal et al. 2012; Colijn and Gardy 2014; Chindelevitch et al. 2021; Barzilai and Schrago 2023). More than twenty conventional tree balance or imbalance indices have been defined (Fischer et al. 2023) yet all have important shortcomings (Lemant et al. 2022; Noble and Verity 2023). Some conventional tree balance indices are defined only for bifurcating trees; others are meaningful only if no nodes have outdegree one. All require adjustments to compensate for correlation with tree size or degree distribution, which hampers the comparison of dissimilar trees. Lack of universality importantly limits practical applications.

We recently introduced a universal tree balance index, denoted $J^{1}$, that has no such flaws. $J^{1}$ is defined for any rooted tree topology and accounts for arbitrary node sizes (which may, for example, correspond to the population sizes of biological types in an evolutionary tree) (Lemant et al. 2022). We proved that $J^{1}$ is robust, in the sense that it is insensitive to small changes in node sizes and to the removal of small nodes. We further showed that this index – which derives from the Shannon entropy – both unites and generalizes the two most popular prior approaches to quantifying tree balance in biology.

Our new index has diverse potential applications. Applied to evolutionary trees, we and others have shown that $J^{1}$ outperforms conventional tree balance indices as a summary statistic for comparing trees inferred from empirical data to computational model predictions (Noble et al. 2022; Feder and Gao 2024). $J^{1}$ also provides promising new ways to infer speciation mode from phylogenies (Freitas et al. 2024) and to predict the outcome of cancer immunotherapy from tumour evolutionary trees (Bozic et al. 2024).

Given any new tree shape index, an important task is to obtain its expected and extremal values under standard tree-generating processes, which can then be used as null-model reference points (Fischer et al. 2023). In Lemant et al. (2022) we obtained analytical approximations to the expected values of $J^{1}$ under the Yule process and the uniform model, and we tested their accuracy numerically for trees with up to 128 leaves. We also showed that the caterpillar tree does not always minimize $J^{1}$ among trees with only zero-sized internal nodes, uniform leaf sizes, and no nodes of outdegree one. Generalizing these results has remained an open problem.

The contributions of this paper are threefold. First, we further establish $J^{1}$ as a universal index of tree balance by identifying fundamental connections to classical results in computer science, related to Huffman coding and self-balancing tree data structures. Second, we prove the accuracy of our expected value approximations for the Yule process and the uniform model. For the Yule process, we derive a new, closer approximation that rapidly converges to the true expectation in the large-tree limit. Finally, we investigate the minimal values of $J^{1}$ in an important special case, obtaining a counter-intuitive result in the large tree limit.

## 2 Results

### 2.1 Preliminary definitions

#### Definition 1 (Rooted tree).

A **rooted tree** T is a connected acyclic graph in which one node is designated the root. Parent-child and ancestor-descendant relationships in a rooted tree are assigned along paths directed away from the root.

#### Definition 2 (Node size and tree magnitude, Lemant et al. (2022)).

We assign to each node i a non-negative **node size**, $w_{i}$. The **magnitude** of a tree T is the sum of its node sizes:

$$S(T)=\sum_{i\in \;V(T)}w_{i},$$

where V(T) is the set of all nodes. To avoid confusion we will not refer to the size of a tree, which is conventionally defined as its node count.

#### Definition 3. (Leafy tree, Lemant et al. (2022))

A **leafy tree** is one with only zero-sized internal nodes.

#### Definition 4. (Node depth and tree height)

We define the **depth** of a node as the number of edges in the shortest path from the root to that node, and the **height** of a tree as its maximum node depth.

#### Definition 5 (Sackin index, Sackin (1972), external path length and internal path length).

The **Sackin index** of rooted tree T is the sum of its leaf depths:

(1) $$I_{S}(T)=\sum_{l\in L(T)}\nu (l),$$

where L(T) is the set of all leaves (terminal nodes) of T, and ν(l) is the depth of leaf l. In computer science this is known as the **external path length**, whereas the **internal path length** is the sum of all internal node depths (Knuth 1997) (p. 400).

#### Definition 6 (Generalized Sackin index, Lemant et al. (2022) or weighted path length).

The Sackin index can be generalized to account for arbitrary node sizes:

(2) $$I_{S,\mathrm{gen}}(T)=\sum_{i\in V(T)}w_{i}\nu (i)=\sum_{i\in \tilde{V}(T)}S_{i}^{*},$$

where $\tilde{V}(T)$ the set of all internal nodes (non-leaves) whose descendants are not all of zero size, and $S_{i}^{*}$ is the magnitude of the subtree rooted at node i, excluding i. If T is a leafy tree in which all leaves have unit size then $I_{S,\mathrm{gen}}(T)=I_{S}(T)$. In computer science, $I_{S,\mathrm{gen}}(T)$ is known as the **weighted path length** (Knuth 1997) (p. 402), denoted |T|.

#### Definition 7 (Binary, bifurcating, full m-ary, and linear trees).

A **binary tree** is a rooted tree in which no node has more than two children. A **full** m-**ary tree** is a rooted tree in which each internal node has exactly m children. The terms **bifurcating** and full binary are also used when m=2, in which case every internal node is associated with a left and a right subtree, of which its two children are the roots. If m=1 then the tree is **linear**.

#### Definition 8 (Cherry).

A tree consisting of only a root and two leaves is a **cherry**.

#### Definition 9 (Caterpillar tree).

A **caterpillar tree** is a bifurcating tree in which every internal node except one has exactly one child leaf.

### 2.2 The universal tree balance index $J^{1}$

In Lemant et al. (2022) we introduced a general class of tree shape indices of the form

(3) $$J(T)=\frac{1}{\sum_{k\in \tilde{V}(T)}g_{k}}\sum_{i\in \tilde{V}(T)}g_{i}W_{i},$$

where $g_{k}$ is a node importance factor and $W_{i}$ is a node balance score. We showed that if the importance factors and balance scores satisfy certain conditions then J can, in a precise sense, be considered a robust, universal tree balance index. Within this general class, we further defined a particular index $J^{1}$ based on a node balance score function $W^{1}$. For every child j of an internal node i, the node balance score $W_{ij}^{1}$ is defined in terms of the normalized Shannon entropy:

(4) $$W_{ij}^{1}=\left\{\begin{array}{ll} -\frac{S_{j}}{S_{i}^{*}}\log_{d^{+}(i)}\frac{S_{j}}{S_{i}^{*}}, & \text{for}\;d^{+}(i)>1\\ 0, & \text{otherwise,} \end{array}\right.$$

where $S_{i}$ is the magnitude of the subtree rooted at node i, including i, and $d^{+}(i)$ is the outdegree (number of children) of i. The tree balance index $J^{1}$ is then defined as a weighted mean of node balance scores by setting $g_{i}=S_{i}^{*}$ and $W_{i}=\sum_{j\in C(i)}W_{ij}^{1}$ :

(5) $$J^{1}(T)=\frac{1}{I_{S,\mathrm{gen}}(T)}\sum_{i\in \tilde{V}(T)}S_{i}^{*}\sum_{j\in C(i)}W_{ij}^{1},$$

where C(i) is the set of children of node i. Note that, for all i∈C(i), we have $0\leq W_{i}\leq 1$ and so $J^{1}$, being a weighted average of $W_{i}$ values, has extrema zero (minimally balanced) and one (maximally balanced).

In Lemant et al. (2022) we proved that, in the case of full m-ary trees, $J^{1}$ is identical to the normalized reciprocal of the generalized Sackin index:

#### Proposition 1 (The leafy tree identity (Lemant et al. 2022)).

*Let* T *be a leafy tree with* $d^{+}(i)=m>1$ *for all internal nodes* i. *Then*

(6) $$J^{1}(T)=\frac{H_{m}(T)S(T)}{I_{S,gen}(T)},$$

*where* $H_{m}(T)=-\sum_{i\in L(T)}\frac{f(i)}{S(T)}\log_{m}\frac{f(i)}{S(T)}$ *is the Shannon entropy (base* m*) of the leaf sizes* f(i) *divided by their sum* S(T). *If additionally the leaves are equally sized then* $J^{1}(T)=n\log_{m}n/I_{S}(T)$.

Moreover, we proved that this property is unique to $J^{1}$ among all indices defined as weighted means of node balance scores. Following Noble and Verity (2023), we will refer to Proposition 1 as the leafy tree identity.

### 2.3 $J^{1}$ unites and generalizes prior notions of tree balance

In computer science, tree balance is effectively a binary property: a tree is considered balanced if its height is sufficiently small, given its leaf count. A precise definition for binary trees is that the height of the left subtree of every node must be no more than one branch longer or shorter than the height of its right subtree (Knuth 1998) (p. 459). In biology, where comparisons between trees are more relevant, it is conventional instead to use a normalized form of the Sackin index or another index to assign balance values on a continuum (Shao and Sokal 1990; Fischer et al. 2023). Subsequent to defining $J^{1}$ in Lemant et al. (2022), we have discovered that our index uniquely connects these two historically separate notions of tree balance.

Key to establishing this connection is the concept of a self-balancing binary search tree. Binary search trees are data structures in which each node is associated with an item of data and information is retrieved by following a path down the tree from the root to the relevant node. The expected time taken to retrieve (or add or remove) data from a binary search tree is thus proportional to the internal path length, and the worst-case time taken is proportional to the tree height. A self-balancing binary search tree is one that automatically adjusts itself to maintain a relatively small height as nodes are added and deleted. Nievergelt and Reingold (1972) introduced one of the most popular types of self-balancing binary search tree, originally called a tree of bounded balance but now more commonly known as a weight-balanced tree (Knuth 1998) (p. 476). Their crucial insight was that the height and the internal and external path lengths can be kept small by ensuring that, for all internal nodes, the ratio of the left subtree leaf count and the right subtree leaf count stays sufficiently close to unity.

The principles underlying the weight-balanced tree concept were presented in Wong and Nievergelt (1973), which derived upper bounds for the internal and external path lengths of binary trees. In proving these results, Wong and Nievergelt (1973) defined what they called the entropy of a node. They consequently defined the average entropy of a tree and stated an identity relating this average entropy to the external path length. The entropy of a node is identical to our node balance score in the special case of full m-ary leafy trees with uniform leaf sizes. Average entropy is (ignoring an erroneous division sign in their definition) equivalent to $J^{1}$ restricted to the same special case. Moreover, the identity of Wong and Nievergelt (1973) is equivalent to the special case of the leafy tree identity (Proposition 1). A seldom cited paper by Nievergelt et al. (1972) implicitly extended these concepts to account for arbitrary node sizes. The universal tree balance index that we call $J^{1}$ was thus defined – and then effectively forgotten – fifty years before we independently rediscovered it.

### 2.4 $J^{1}$ is maximized by Huffman coding

Huffman coding provides another way to understand the equivalence between minimizing binary tree path length and maximizing $J^{1}$.

#### Definition 10 (Huffman coding, Huffman (1952)).

**Huffman coding** is a method for constructing a leafy binary tree with minimal weighted path length, given a set of leaves with arbitrary sizes $\alpha_{1},\ldots ,\alpha_{n}>0$. We begin with the set $B=\left\{t_{1},\ldots ,t_{n}\right\}$, where each $t_{i}$ is a tree comprising exactly one node of size $\alpha_{i}$. Without loss of generality, let $t_{1}$ and $t_{2}$ be the two smallest magnitude trees in B. We then combine $t_{1}$ and $t_{2}$ by attaching them to a root of size zero to obtain $B=\left\{t_{1,2},\ldots ,t_{n}\right\}$. We repeat this process until B comprises a single tree $t_{1,2,\ldots ,n}$, called the **Huffman tree**.

#### Proposition 2.

*The Huffman tree maximizes* $J^{1}$ *on bifurcating leafy trees for a given set of leaf sizes*.

*Proof* By the leafy tree identity, the Huffman method maximizes $J^{1}$ as it minimizes the weighted path length. □

### 2.5 Expected values of $J^{1}$ under standard null models

For applications in evolutionary biology, it is important to know the expected values of a balance index under simple null models. These expected values can then be compared with values obtained for trees inferred from empirical data, to test hypotheses about the nature of evolution (Heard 1992; Mooers and Heard 1997; McKenzie and Steel 2000; Aldous 2001; Steel and McKenzie 2001). The simplest and most widely studied null models are the uniform model and the Yule model (or pure birth process), both of which generate bifurcating unlabelled trees.

#### Definition 11 (Yule model, Yule (1925)).

**Fig. 1:**
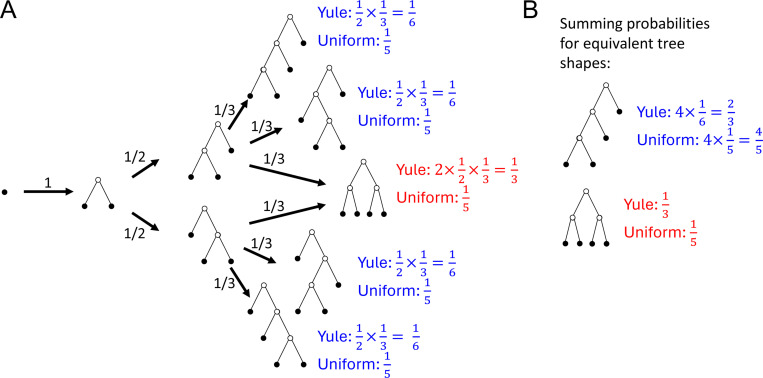
**A:** Tree generation under the Yule model. Each four-leaf tree is labelled with the probabilities it is assigned by the Yule and uniform models. **B:** Tree shape probabilities are obtained by summing over trees that can be transformed into one another by rotating branches, such as the four asymmetric trees in panel A (blue labels).

The **Yule model** is associated with the following process: Starting with a cherry, repeatedly replace one leaf, chosen uniformly at random, with a cherry, so that the number of leaves increases by one at each step (Figure 1A). For a given number of leaves, each tree is assigned a probability proportional to the number of ways it can be generated by this process.

#### Definition 12 (Uniform model, Rosen (1978)).

Under the **uniform model**, every bifurcating tree on n leaves is assigned the probability $n{\left(\begin{array}{c} 2n-2\\ n-1 \end{array}\right)}^{-1}$, the inverse of the number of distinct trees on n leaves.

Tree balance indices assign equal values to trees that can be transformed into one another by rotating branches. We can therefore sum probabilities over all trees with equivalent shapes (Figure 1B). Since the Yule and uniform models do not assign node sizes, we will consider only leafy trees with uniform leaf sizes. In systematic biology, such trees can be interpreted as cladograms in which leaves represent extant types and internal nodes represent extinct common ancestors (Podani 2013).

**Fig. 2:**
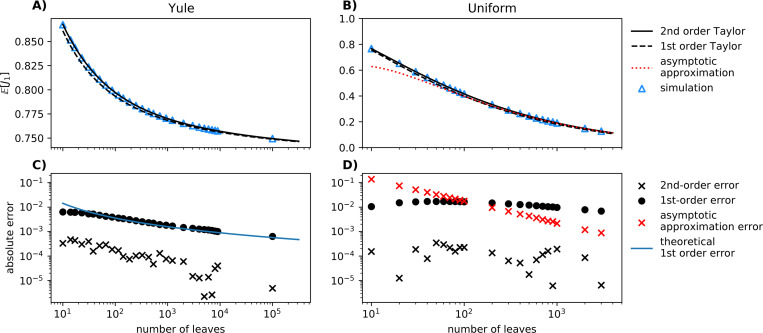
$E\left[J^{1}\right]$ approximations along with associated errors. **Top row**: Blue triangles show the sample mean of $J^{1}$ under the Yule **(A)** and uniform **(B)** tree generation processes. 1st and 2nd order Taylor approximations are shown for both processes. The 1st order approximation also corresponds to a harmonic mean-expectation approximation. In the uniform model, an approximation based on the asymptotic distribution of $X_{n}=\frac{I_{S}}{n^{3/2}}$ is shown in red. At each of the designated leaf counts, we sample 10^6^ trees under the Yule or uniform process. We use the straightforward recursion equation to sample Yule trees (Eq. A11), and we employ the random bracket sequence method described in Atkinson and Sack (1992) to sample uniform binary trees. **Bottom row**: The errors of each approximation type (Yule **(C)** and uniform **(D)**) are shown with respect to the tree size. The blue line indicates the theoretically expected error. For further details of the approximations see Appendix A.2.

The leafy tree identity implies that the expected value of $J^{1}$ for full m-ary leafy trees with n equally sized leaves is

(7) $$E\left(J^{1}\right)=E\left(\frac{n\;\log_{2}n}{I_{S}}\right)=\frac{n\;\log_{2}n}{ℍ\left(I_{S}\right)},$$

where $ℍ\left(I_{S}\right)=1/E\left(1/I_{S}\right)$ is the harmonic mean of the Sackin index. Obtaining the expected value of $J^{1}$ for such trees under any model is therefore equivalent to obtaining the harmonic mean of the Sackin index under the same model. Although we have yet to find a closed-form expression for the harmonic mean of $I_{S}$ under either of the standard null models, we have obtained exact expected values of $J^{1}$ for n≤11 (Yule model) and n≤10 (uniform model) by iteratively generating all possible n-leaf trees (Tables A1 and A2; black crosses in Figure 2).

We can also approximate the expected value of $J^{1}$ by using the arithmetic mean to approximate the harmonic mean: $ℍ\left(I_{S}\right)\approx E\left(I_{S}\right)$. Under the Yule and uniform models, the expected values of the Sackin index for trees on n leaves are respectively

(8) $$E_{Y}\left(I_{S}\right)=2n\sum_{i=2}^{n}\frac{1}{i}=2n\left(H_{n}-1\right),\;E_{U}\left(I_{S}\right)=n\left(\frac{(2n-2)!!}{(2n-3)!!}-1\right),$$

where $H_{n}$ is the nth harmonic number and k!! is the double factorial k!!=k(k−2)(k−4)… (Kirkpatrick and Slatkin 1993; Mir et al. 2013). Hence

(9) $$E_{Y}\left(J^{1}\right)\approx \frac{\log_{2}n}{2\left(H_{n}-1\right)},\;E_{U}\left(J^{1}\right)\approx \frac{(2n-3)!!\log_{2}n}{(2n-2)!!-(2n-3)!!}.$$

The error in each approximation is the Jensen gap

(10) $$J(n)=E\left(J^{1}\right)-\frac{n\log_{2}n}{E\left(I_{S}\right)}.$$

In Lemant et al. (2022) we obtained numerical results, based on samples of randomly generated trees, suggesting that J(n) is relatively small for n≤128.

A result of Liao and Berg (2019) leads to the following more rigorous and precise new results:

#### Proposition 3.

*For bifurcating leafy trees with n equally sized leaves:*

1. *For the Yule model*, J(n)<0.008 *for all* n, *and* J(n)→0 *as* n→∞, *which implies that, asymptotically*, $J^{1}\approx \frac{1}{2\ln2}\approx 0.72$ *(in the sense that the distribution converges to this value as* n→∞*)*.
2. *For the uniform model*, $J(n)<4/\left(3\pi e^{2}\right)\approx 0.057$ *for all* n.

*Proof* See Appendix A.1. □

The first part of Proposition 3 can be generalized to the Yule process for full m-ary trees (see Appendix A.2.1).

The errors in our first-order approximation (black dots in Figure 2C and 2D) lead us to conjecture further that J(n) for the uniform model is never more than 0.02 and approaches zero as n→∞. The gap is larger for the uniform model than for the Yule model because $I_{S}$ has greater variance (Figure A1).

The approximation $E\left[\frac{1}{I_{S}}\right]\approx \frac{1}{E\left[I_{S}\right]}$ can also be interpreted as the moment of a first-order Taylor approximation of the function $f(x)=\frac{1}{x}$ composed with the random Sackin index. Using this approach, we obtain more precise approximate asymptotic error expressions in the Yule case and find that J(n) decays at an asymptotic approximate rate of ${\left(\ln n\right)}^{-2}$. We can further perform a second-order Taylor expansion:

(11) $$E\left[J^{1}\right]\approx \frac{n\;\log_{2}n}{E\left[I_{S}\right]}+\frac{n\;\log_{2}n}{E{\left[I_{S}\right]}^{3}}V\left[I_{S}\right],$$

where V denotes the variance. This approximation decays at a faster rate ${\left(\ln n\right)}^{-3}$. In the uniform model in Figure 2, we empirically observe that equation 11 is an accurate approximation of $E\left[J^{1}\right]$. An asymptotic approximation based on the asymptotic distribution of $X_{n}=\frac{I_{S}}{n^{3/2}}$ in the uniform model is conjectured to decay at polynomial rate (red crosses in Figure 2d). All these results are derived in Appendix A.2.

### 2.6 Least balanced leafy trees with equally sized leaves

**Fig. 3:**
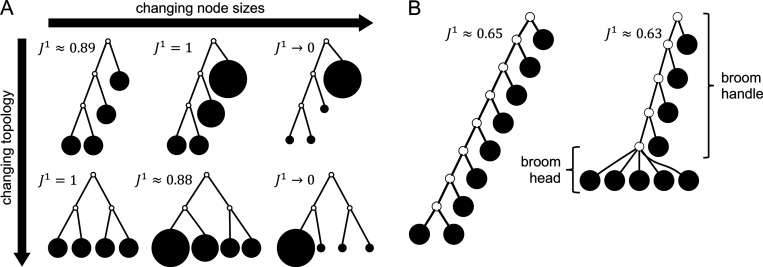
**A:** By varying relative node sizes, any non-linear tree can be made maximally or minimally balanced according to $J^{1}$, as illustrated here with a leafy four-leaf caterpillar tree (top row) and a leafy four-leaf tree with symmetric topology (bottom row). The leaf sizes in the first column are all equal and in the second column are 4,2,1,1. **B:** The leafy caterpillar and minimally balanced leafy broom tree on nine equally sized leaves. In all trees, open circles represent internal nodes of size zero.

We now turn from expected to minimal values. We previously proved that $J^{1}$ attains its minimal value 0 if and only if the tree is linear, and its maximal value 1 if and only if each internal node splits its descendants into at least two subtrees of equal magnitude (Lemant et al. 2022). Because $J^{1}$ accounts for node sizes, any non-linear tree, regardless of topology, can be made arbitrarily unbalanced $\left(J^{1}\to 0\right)$ or arbitrarily balanced $\left(J^{1}=1\right)$ by adjusting its relative node sizes (Figure 3A). It is therefore trivial to find the most and least balanced trees across all possible node sizes. The most important open questions concern the extremal values of $J^{1}$ when the node degree and node size distributions are somehow constrained.

The simplest interesting case is that of leafy trees with equally sized leaves. Among bifurcating trees with a given number of leaves, the caterpillar tree maximizes Sackin’s index (Fischer 2021). Consequently, the leafy tree identity implies that, among all bifurcating leafy trees with a given number of equally sized leaves, the caterpillar tree minimizes $J^{1}$. However, we previously presented a counterexample showing that the caterpillar tree does not always minimize $J^{1}$ when larger outdegrees are permitted (Lemant et al. 2022). Specifically, we showed that, among all leafy trees with six equally sized leaves and no nodes of outdegree 1, the least balanced tree according to $J^{1}$ is not a caterpillar but belongs to a superset of caterpillar trees that we shall call broom trees.

#### Definition 13 (Broom tree).

A **broom tree** is such that every internal node except the most distant from the root has outdegree 2. The **broom head** is the star subtree rooted at the lowest internal node; the remainder of the tree is the **handle** (Figure 3B).

It is straightforward (see Appendix A.3) to derive a general expression for $J^{1}$ for leafy broom trees with n equally sized leaves, of which k are in the head:

(12) $$J_{B}^{1}(n,k,1):=\frac{2\left(n\log_{2}n-k\log_{2}k+k\right)}{\left(n+k)(n-k+1\right)}.$$

By varying the value of k in this expression, we can verify, for example, that the caterpillar is not the minimally balanced leafy broom tree with nine equally sized leaves (Figure 3B). The following proposition generalizes this result.

#### Proposition 4.

*Among leafy trees with* n *equally sized leaves and no nodes of outdegree 1, the caterpillar minimizes* $J^{1}$ *if and only if* n≤4.

*Proof* If n=2 then the proposition holds as the caterpillar is the only tree, and for n=3 it holds because the caterpillar is less balanced than the star tree, which is the only alternative. From Equation 12 for n>3 we find that $J_{B}^{1}(n,2,1)<J_{B}^{1}(n,3,1)$ if and only if

(13) $$4n\log_{2}n+3\left(1-\log_{2}3\right)(n+2)(n-1)>0.$$

Given n>3, this inequality is satisfied if and only if n is less than approximately 4.17. As n must be a positive integer, the only valid solution is n=4. □

The non-optimality of caterpillar trees can be understood by considering how the node balance scores and weights change when we remove the second lowest internal node of a caterpillar tree and reattach its child leaf to the lowest internal node. First, the normalizing factor of $J^{1}$ (which is identical to Sackin’s index) decreases. Second, while the internal nodes that remain in the broom handle have the same balance scores and weights as before the change, the weight assigned to the root of the broom head, which remains maximally balanced, increases due to its additional leaf. Although assigning more weight to the broom head and reducing the normalizing factor both increase $J^{1}$, these effects are counteracted by the removal of the handle’s most balanced node. The latter effect dominates when the leaf count is greater than 4 (see Appendix A.4 for a more detailed explanation).

The analysis of broom trees illustrates the general principle that when two trees differ in outdegree distribution, $J^{1}$ accounts for differences in node balance scores and weights as well as in node depths. By contrast, if two leafy trees are both full m-ary and have the same number of leaves and the same leaf size distribution, then the difference in $J^{1}$ is solely determined by the difference in Sackin’s index (because, by Proposition 1, the weighted sum of the node balance scores must be the same for both trees).

We further make the following conjecture, which we have exhaustively verified for trees with 12 or fewer leaves.

#### Conjecture 5.

*Among leafy trees with a given number of equally sized leaves and no nodes of outdegree 1, the tree that minimizes* $J^{1}$ *is a broom tree*.

### 2.7 Least balanced leafy broom trees in the large n limit

The obvious question arising from Proposition 4 and Conjecture 5 is this: Which leafy broom tree is the least balanced for a given leaf count? To examine whether the answer is sensitive to our assumption of equal leaf sizes, we will slightly relax this assumption by considering leafy broom trees in which all leaves in the head have the same size, and all leaves in the handle have the same size, while allowing these two sizes to differ. The task is then to investigate $k^{*}=\arg\min_{2\leq k\leq n}J_{B}^{1}(n,k,p)$, where n is the total number of leaves, k is the number of leaves in the head, p>0 is the size of each leaf in the head, relative to the size of each leaf in the handle, and

(14) $$J_{B}^{1}(n,k,p):=\frac{2\left[kp+(kp+n-k)\log_{2}(kp+n-k)-kp\log_{2}(kp)\right]}{\left(2kp+n-k)(n-k+1\right)}.$$

Note that, although $k^{*}$ depends on n and p, we will tend to avoid writing $k_{n,p}^{*}$ to simplify our notation.

**Fig. 4:**
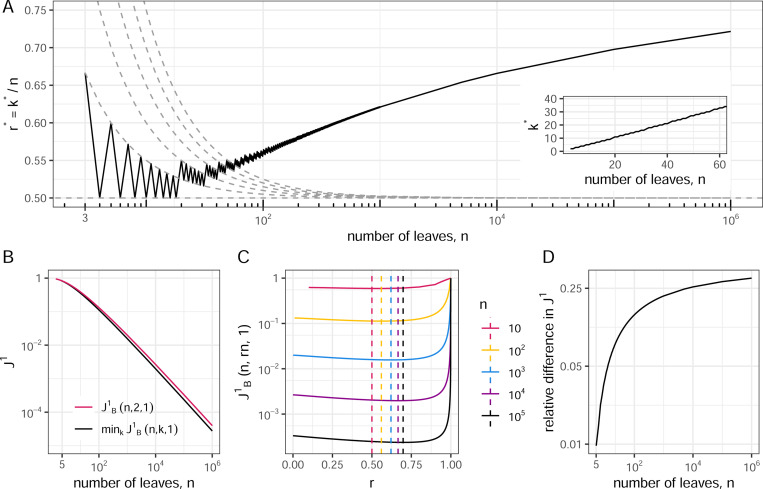
Numerical results for least balanced leafy broom trees with equally sized leaves. **A:** Values of r=k/n (main plot) and k (inset) that minimize $J_{B}^{1}(n,k,1)$. The discrete values are connected by solid black lines to guide the eye. Dashed grey curves in the main plot show $r^{*}=\frac{n+a}{2n}$ for a∈{0,1,2,3,4,5}. These curves intersect with the $r^{*}$ values of trees for which $k^{*}=\frac{n+a}{2}$. **B:** Exact $J_{B}^{1}$ values for caterpillar trees and minimally balanced leafy broom trees with equally sized leaves, as a function of n. **C:** $J_{B}^{1}(n,rn,1)$ versus r for different values of n. The dashed lines are at the values of r that minimize $J^{1}$. **D:** The relative difference in $J^{1}$ values between the minimally balanced broom tree and the caterpillar tree, as a function of n.

Among leafy broom trees with equally sized leaves (that is, p=1), a numerical investigation of Equation 14 reveals that that $r^{*}=k^{*}/n$ is never less than $\frac{1}{2}$ and oscillates while generally increasing with n (Figure 4A). We can understand the upward trend in $r^{*}$ by considering the large tree limit.

#### Proposition 6.

*Treating* $J_{B}^{1}$ *as a function of* n, *the following hold:*

*If* $0<p\leq \frac{1}{2}$ *then there exists* $N_{p}$ *such that for all* $n>N_{p}$, $k^{*}=2$. *Hence*

(15) $$\underset{2\leq k\leq n}{\min}J_{B}^{1}(n,k,p)=J_{B}^{1}(n,2,p)∼\frac{2\log_{2}n}{n}.$$

*If* $p>\frac{1}{2}$ *then*

(16) $$1-r_{n}^{*}∼\frac{p\sqrt{2}}{\sqrt{(2p-1)\log_{2}n}},$$

(17) $$\underset{2\leq k\leq n}{\min}J_{B}^{1}(n,k,p)∼\frac{\log_{2}n}{np}.$$

*Proof* See Appendix A.5. □

Proposition 6 implies that the caterpillar is the least balanced broom tree when $p\leq \frac{1}{2}$ and n is sufficiently large. In contrast if $p>\frac{1}{2}$ then, for sufficiently large n, the least balanced broom tree has most of its leaves on the head, with relatively few on the handle. In the case of equally sized leaves (p=1), the absolute difference in $J^{1}$ values between the minimally balanced broom and the caterpillar is always small and decreases rapidly as the number of leaves increases (Figure 4B,C), yet Proposition 6 implies that the relative difference approaches $\frac{1}{2}$ (Figure 4D).

### 2.8 Least balanced leafy broom trees in the general case

**Fig. 5:**
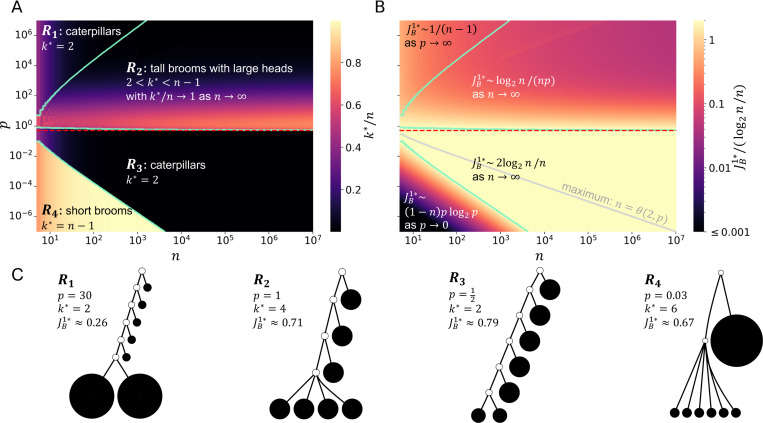
Least balanced broom trees in n−p space. **A:** For n>4, there are four cases of $k^{*}=\arg\;\min_{2\leq k\leq n}J_{B}^{1}(n,k,p)$, separated here by turquoise curves. The case $k^{*}=2$ corresponds to caterpillar trees. The middle curve approaches $p=\frac{1}{2}$ (red dashed line) as n→∞. **B:** $J_{B}^{1*}=\min_{2\leq k\leq n-1}J_{B}^{1}(n,k,p)$ relative to $\log_{2}n/n$. Each region is labelled with the corresponding asymptotic behaviour of $J_{B}^{1*}$. The grey curve at n=θ(2,p) is where $J_{B}^{1*}$ is maximal (the formula for θ(k,p) is given in Proposition 10). **C:** A representative least balanced broom tree for each of the four cases when n=7. Open circles represent internal nodes of size zero.

For trees with fewer leaves the picture is more complicated. If n=3 then $k^{*}=2$ for all p>0. For n=4 there are two cases: $k^{*}=2$ and $k^{*}=n-1=3$, depending on p. For n>4 there are four cases corresponding to regions of n−p space that we will label $R_{1}$ to $R_{4}$ (Figure 5A, 5C). In the non-adjacent $R_{1}$ and $R_{3}$, we have $k^{*}=2$, which means that the least balanced trees in these regions are caterpillars. In $R_{2}$, we find $2<k^{*}<n-1$ with $k^{*}/n\to 1$ as n→∞, so that the least balanced trees have both long handles and large heads. In $R_{4}$, $k^{*}=n-1$, corresponding to trees of height 2.

To locate the regional boundaries, we first note that Proposition 6 implies that the curve separating regions $R_{2}$ and $R_{3}$ approaches $p=\frac{1}{2}$ as n→∞. Proposition 4 also tells us that p>1 for all $(n,p)\in R_{1}$ and p<1 for all $(n,p)\in R_{3}$. The following proposition further establishes that $p>\frac{1}{2}$ for all $(n,p)\in R_{2}$ and $p<\frac{1}{2}$ for all $(n,p)\in R_{4}$. Hence, in summary, the $R_{2}-R_{3}$ boundary is bounded below by $p=\frac{1}{2}$, bounded above by p=1, and approaches the lower bound as n→∞.

#### Proposition 7.

*If* $p=\frac{1}{2}$ *then* $k^{*}=2$ *for all* n≥3.

*Proof* See Appendix A.6. □

Just as we did for the large-*n* limit, we can also describe the nature of $J^{1}$ for the least-balanced broom tree in the large- and small-p limits.

#### Proposition 8.

*For any fixed* n≥3:

1. *As* p→∞, $J_{B}^{1}\left(n,k^{*},p\right)∼\frac{1}{n-1}$.
2. *As* p→0, $J_{B}^{1}\left(n,k^{*},p\right)∼p(1-n)\log_{2}p$.

*Proof* See Appendix A.7. □

Lastly, we show that $R_{1}$ and $R_{4}$ contain trees with arbitrarily large leaf counts:

#### Proposition 9.

*The boundaries between the* $R_{3}$ *and* $R_{4}$ *regions, and between the* $R_{1}$ *and* $R_{2}$ *regions, are asymptotically* $p=2/n^{2}$ *and* $p=n^{2}\log_{2}n/3$, *respectively*.

*Proof* See Appendix A.8. □

The following results shed further light on how, for a given number of leaves, the $J^{1}$ value of the least balanced broom tree – that is, $\min_{2\leq k\leq n-1}J_{B}^{1}(n,k,p)=J_{B}^{1}\left(n,k^{*},p\right)$ (abbreviated to $J_{B}^{1*}$ in Figure 5B) – varies with p.

#### Proposition 10.

*For all* n≥3 *and* k∈{2,…,n−1}, $J_{B}^{1}(n,k,p)$ *as a function of* p>0 *is strictly decreasing if* n>θ(k,p) *and strictly increasing if* n<θ(k,p), *where* $\theta (k,p)=\frac{2}{kp}+k(1-p)$.

*Proof* Since n>k,

$$\begin{array}{l} \text{sign}\left(\frac{\partial J_{B}^{1}}{\partial p}(n,k,p)\right)=\text{sign}\left(\frac{2k(n-k)\left(1-\log_{2}[kp(n-k(1-p))]\right)}{{\left(2kp+n-k\right)}^{2}(n-k+1)}\right)\\ \;=\text{sign}(\theta (k,p)-n). \end{array}$$

□

#### Proposition 11.

*For all* n≥3, $\min_{2\leq k\leq n}J_{B}^{1}(n,k,p)$ *as a function of* p>0 *is strictly decreasing if* n>θ(2,p) *and strictly increasing if* n<θ(2,p), *where* $\theta (2,p)=\frac{1}{p}+2(1-p)$.

*Proof* See Appendix A.9. □

#### Corollary 11.1.

*For all* n≥3,

$$\underset{2\leq k\leq n}{\min}J_{B}^{1}(n,k,p)\leq \frac{2\left(2-\log_{2}\left(\sqrt{n^{2}-4n+12}-n+2\right)\right)}{n-1},$$

*with equality if and only if* n=θ(2,p).

*Proof* See Appendix A.10. □

The curve n=θ(2,p), where $\min_{2\leq k\leq n}J_{B}^{1}(n,k,p)$ is maximal, is shown in Figure 5B (solid grey curve). The three panels of Figure 5 together summarize all the main results of this section.

## 3 Discussion

The aims of this paper were to explore mathematical properties of the $J^{1}$ index and to solidify its status as a universal tree balance index. By extending past results and uncovering new connections, we have shown that $J^{1}$ unifies the notions of tree balance from biology and computer science. We have set the historical record straight by explaining how the “average entropy of a tree” definition of Wong and Nievergelt (1973) is equivalent to a special case of our $J^{1}$ definition. Yet whereas, throughout the five decades before we independently rediscovered it, this index was regarded merely as an accessory to defining weight-balanced trees, we instead contend that $J^{1}$ is useful in its own right as the best index for quantifying tree balance (Lemant et al. 2022; Noble et al. 2022; Noble and Verity 2023).

While closed-form expressions remain elusive, we have proven the accuracy of simple approximations to the expected values of $J^{1}$ for the two most important tree generating models. These results put $J^{1}$ on the same footing as the conventional Sackin and Colless tree balance indices, for which the corresponding expected values have been derived previously (Kirkpatrick and Slatkin 1993; Blum et al. 2006; Mir et al. 2013). The errors in our approximations are small enough as to be negligible in many practical applications. When this is not the case, statistical methods can employ the maximum and minimum bounds on the true expected values (Figure 2).

We have also investigated minimal values of $J^{1}$ in the interesting special case of broom trees (in which every internal node except the most distant from the root has outdegree 2). A motivation for this investigation is that every tree balance index must, by definition, attain its extremal values only on trees that can reasonably be described as extremely balanced or extremely imbalanced. Also, knowing the tree types for which an index attains its maximum and minimum values aids interpretation of the index, by providing reference points to which other trees can be compared. Given these motivations, we have focussed on leafy broom trees, which provide us with a relatively simple yet non-trivial special case for investigating minimal $J^{1}$ values. Broom trees include caterpillar trees, which are the least-balanced bifurcating trees according to popular conventional tree balance indices (Fischer 2021; Coronado et al. 2020).

We have thus shown that, although caterpillar trees minimize $J^{1}$ among leafy bifurcating trees with equally sized leaves, they are sub-optimal when outdegrees greater than two are permitted. The least balanced broom tree in the more general case has a long handle and a large head (containing at least half the leaves). Nevertheless, most broom trees (all but those with extremely large heads) have relatively similar $J^{1}$ values, which decrease rapidly as the number of leaves increases. By relaxing the assumption of equal leaf sizes, we have further shown that a small change in relative leaf sizes can dramatically change the topology of the least balanced broom tree. In particular, if the leaves in the broom head are moderately smaller than those in the handle then the least balanced broom tree is a caterpillar.

Is it at all problematic that caterpillar trees do not always minimize $J^{1}$? Does this property make $J^{1}$ somehow different from other tree balance indices? We argue that the answer is no in both cases. It is true that caterpillar trees minimize Sackin’s index among all trees with a given number of leaves and no nodes of outdegree one (Fischer 2021). But this property is meaningless because it is meaningless to compare Sackin’s index values for trees with different outdegree distributions (Shao and Sokal 1990; Lemant et al. 2022). Otherwise, for example, the symmetrical bifurcating tree on four leaves would be considered less balanced than the four-leaved star tree. The same restriction applies to the total cophenetic index (Mir et al. 2013). The other popular conventional tree balance index, Colless’ index, is defined only for bifurcating trees (Colless 1982). Hence there is no prior “correct” answer as to whether a given non-bifurcating broom tree is more or less balanced than the caterpillar with the same number of leaves. Our index provides a reasonable, consistent solution to this problem.

An important goal for future research is to obtain the expected values of $J^{1}$ for more complicated yet more biologically realistic models. These include models that generate non-leafy trees, such as the clone trees that are central to cancer evolution research (Noble et al. 2022; Feder and Gao 2024). Our recent generalization of $J^{1}$ to account for branch lengths (Noble and Verity 2023) motivates the study of models that generate non-uniform branch lengths. It should also be possible to investigate the expected values of extensions of $J^{1}$ that apply to phylogenetic networks, as was recently done for Sackin’s index (Fuchs and Gittenberger 2025).

The methods we have applied here to finding least-balanced broom trees can be readily adapted to other special tree topologies. In particular, given that Conjecture 5 applies only in the case of equally-sized leaves, it will be worthwhile to examine minimal $J^{1}$ values when the node size distribution is such that broom trees clearly do not minimize $J^{1}$. For example, every broom tree in region $R_{4}$ of Figure 5 has a higher $J^{1}$ value than the leafy star tree with the same number of leaves and the same leaf size distribution.

In conclusion, our results strengthen the case for $J^{1}$ as the most useful cross-disciplinary index of tree balance and provide a firm foundation for using $J^{1}$ instead of conventional indices to compare and categorize empirical trees.

## Supplementary Material

Supplement 1

## Data Availability

All data created for this study can be readily reproduced using our mathematical methods, with the exception of the data presented in Tables A1 and A2. Efficient R code for calculating $J^{1}$ is available at https://zenodo.org/records/5873857.
