# Supplementary material for: Expected and minimal values of a universal tree balance index
Source: ArXiv. 2025 Jul 11:arXiv:2507.08615v1. Preprint. [Version 1] (PMC12265486)
Supplement: Supplement 1 [file NIHPP2507.08615v1-supplement-1.pdf]

Appendix A Additional proofs and derivations

A.1 Proof of Proposition 3

Proof Our proof of Proposition 3 relies on a recent result of Liao and Berg (2019).

Theorem 12 (Liao and Berg (2019)). *Let X be a one-dimensional random variable with mean μ, and $P(X \in (a,b)) = 1$, where $\infty \leq a < b \leq \infty$. If $f(x)$ is a twice differentiable function on (a,b), and*

$$h(x;\nu) = \frac{f(x) - f(\nu)}{(x-\nu)^2} - \frac{f'(\nu)}{x-\nu},$$

then

$$\inf_{x\in(a,b)} \{h(x;\mu)\}\mathrm{Var}(X) \leq \mathbb{E}[f(X)] - f(\mathbb{E}[X]) \leq \sup_{x\in(a,b)} \{h(x;\mu)\}\mathrm{Var}(X). \tag{A1}$$

Part i

Let μ_Y be the expected value of the Sackin index under the Yule process for trees on n leaves, let $f(x) = \frac{n\log_2 n}{x}$, and define

$$h(x;\mu_Y) = \frac{f(x) - f(\mu_Y)}{(x-\mu_Y)^2} - \frac{f'(\mu_Y)}{x-\mu_Y} = \frac{n\log_2 n}{x\mu_Y^2}. \tag{A2}$$

Theorem 12 then implies

$$\frac{n\log_2 n}{\frac{(n-1)(n+2)}{2}\mu_Y^2}\mathrm{Var}_Y(I_S) \leq \mathbb{E}[J^1] - \frac{n\log_2 n}{\mathbb{E}[I_S]} \leq \frac{n\log_2 n}{\mu_Y^2 n\log_2 n}\mathrm{Var}_Y(I_S), \tag{A3}$$

where the supremum and infimum of $h(x,\mu)$ are the extremal values of the Sackin index on bifurcating trees (Fischer 2021). The expectation of the Sackin index under the Yule process is given in Equation 8, and its variance as derived by Cardona et al. (2013) is

$$\mathrm{Var}_Y(I_S) = 7n^2 - 4n^2\sum_{i=1}^{n}\frac{1}{i^2} - 2n\sum_{i=1}^{n}\frac{1}{i} - n. \tag{A4}$$

Substituting these expressions into Equation A3, we obtain the limits

$$\begin{aligned}\frac{n\log_2 n}{\frac{(n-1)(n+2)}{2}\mu_Y^2}\mathrm{Var}_Y(I_S) &\overset{n\to\infty}{\sim} \frac{\log_2 n\left(7n^2 - 4n^2\sum_{i=1}^{n}\frac{1}{i^2} - 2n\sum_{i=1}^{n}\frac{1}{i} - n\right)}{2n^3\left(\sum_{i=2}^{n}\frac{1}{i}\right)^2}\\ &\overset{n\to\infty}{\sim} \frac{\left(7 - \frac{2\pi^2}{3}\right)n^2\log_2 n}{2n^3(\ln n)^2}\\ &\overset{n\to\infty}{\sim} \frac{21 - 2\pi^2}{6n\ln 2\ln n} \to 0\end{aligned}$$

for the lower bound on the gap, and

$$\begin{aligned}\frac{n\log_2 n}{\mu_Y^2 n\log_2 n}\mathrm{Var}_Y(I_S) &\overset{n\to\infty}{\sim} \frac{7n^2 - 4n^2\sum_{i=2}^{n}\frac{1}{i^2} - 2n\sum_{i=2}^{n}\frac{1}{i} - n}{4n^2\left(\sum_{i=2}^{n}\frac{1}{i}\right)^2}\\ &\overset{n\to\infty}{\sim} \frac{\left(7 - 4\left(\frac{\pi^2}{6} - 1\right)\right)n^2}{4n^2(\ln n)^2}\end{aligned}$$

$$\overset{n\to\infty}{\sim} \frac{7 - 4\left(\frac{\pi^2}{6} - 1\right)}{4(\ln n)^2} \to 0$$

for the upper bound on the gap. The upper bound reaches a maximum of approximately 0.00790 at $n = 13$ and the lower bound reaches a maximum of approximately 0.00499 at $n = 8$.

Part ii

Let μ_U be the expected value of the Sackin's index under the uniform model for trees on n leaves, and $f(x) = \frac{n \log_2 n}{x}$, and define

$$h(x; \mu_U) = \frac{f(x) - f(\mu_U)}{(x - \mu_U)^2} - \frac{f'(\mu_U)}{x - \mu_U} = \frac{n \log_2 n}{x\mu_U^2}. \tag{A5}$$

Theorem 12 then implies

$$\frac{n \log_2 n}{\frac{(n-1)(n+2)}{2}\mu_U^2} \mathrm{Var}_U(I_S) \le \mathbb{E}[J^1] - \frac{n \log_2 n}{\mathbb{E}[I_S]} \le \frac{n \log_2 n}{\mu_U^2 n \log_2 n} \mathrm{Var}_U(I_S), \tag{A6}$$

analogously to Equation A3. The expectation and variance of Sackin's index under the uniform model are (Cardona et al. 2013):

$$\mathbb{E}_U(I_S) = \frac{4^{n-1} n!(n-1)!}{(2n-2)!} - n, \tag{A7}$$

$$\mathbb{V}_U(I_S) = n\frac{10n^2 - 3n - 1}{3} - \frac{(n+1)n}{2}\frac{(2n-2)!!}{(2n-3)!!} - n^2\left(\frac{(2n-2)!!}{(2n-3)!!}\right)^2. \tag{A8}$$

In the limit $n \to \infty$, we obtain:

$$\mathbb{E}_U(I_S) \overset{n\to\infty}{\sim} \sqrt{\pi} n^{3/2}$$
$$\mathbb{V}_U(I_S) \overset{n\to\infty}{\sim} \left(\frac{10}{3} - \pi\right) n^3.$$

It follows that the lower bound of $\mathbb{E}_U(J^1) - \frac{n \log_2 n}{\mathbb{E}_U(I_S)}$ goes to 0 as fast as $\frac{\ln n}{n}$, while the upper bound increases very slowly to the limit $\frac{10}{3\pi} - 1 \approx 0.061$ as $n \to \infty$, as a consequence of high variance in the uniform model (Figure A1). □

A.2 Approximations of $\mathbb{E}[J^1]$

Let $f(x) = \frac{1}{x}$. We are interested in an approximation of such a function in terms of polynomial-order terms in x, and hence it is natural to consider its kth-order Taylor approximation about $\mu > 0$:

$$f(x) = \sum_{i=0}^{k} (-1)^i \frac{1}{\mu^{i+1}} (x - \mu)^i + R_k(x).$$

Here $R_k(x)$ is the error:

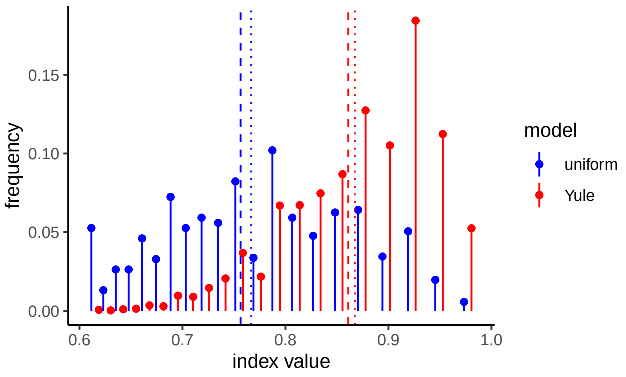

Fig. A1 Exact distributions of J^1 values for 10-leaf trees generated under the Yule process (red) and the uniform model (blue), illustrating that the latter has higher variance. Dashed lines represent $\mathbb{E}(J^1)$ and dotted lines $\frac{n \log_2 n}{\mathbb{E}(I_S)}$.

$$R_k(x) = \frac{f^{(k+1)}(z(x))}{(k+1)!}(x-\mu)^{k+1} = (-1)^{k+1}\frac{(x-\mu)^{k+1}}{z^{k+2}(x)} \tag{A9}$$

where $z(x)$ is a value between x and μ. Composing f with the random variable I_S representing the Sackin index of a randomly generated tree with n leaves, letting $\mu = \mathbb{E}[I_S]$, and taking expectations, we obtain

$$\mathbb{E}\left[\frac{J^1}{n \log_2 n}\right] = \mathbb{E}[f(I_S)] = \frac{1}{\mathbb{E}[I_S]} + \sum_{i=2}^{k}(-1)^i \frac{1}{\mu^{i+1}}\mathbb{E}[(I_S-\mu)^i] + \mathbb{E}[R_k(I_S)], \tag{A10}$$

where the first equality is due to the leafy-tree identity. Thus, the goal is to examine the asymptotic behavior of $\mathbb{E}[R_k(I_S)]$ as n goes to infinity. To generate random trees, we can examine either the Yule or the uniform process. We denote $I_{S,k}$ the random variable corresponding to the Sackin index of either a Yule or uniform tree with k leaves. The subscript k is omitted when the number of leaves is clear from context.

A.2.1 Yule

Under the Yule process, I_S satisfies the following recurrence (Blum and François 2005):

$$I_{S,n} \overset{d}{=} I_{S,V} + I_{S,n-V} + n, \tag{A11}$$

where V is a discrete uniform random variable with support $\{1, \ldots, n-1\}$, $I_{S,V}, I_{S,n-V}$ are independent, conditional on V, and $\overset{d}{=}$ denotes equality in distribution. In fact it is easy to generate simulations of I_S using Equation A11.

It is known that the distribution of $\frac{I_S-\mathbb{E}[I_S]}{n}$ is the same as that of $\frac{Q_n-\mathbb{E}[Q_n]}{n}$, where Q_n is the number of steps required for a Quicksort of a random n-length string. This follows from $I_S \overset{d}{=} Q_n + 2(n-1)$ (Iliopoulos 2015). Additionally, $\frac{Q_n-\mathbb{E}[Q_n]}{n}$ converges in distribution to a random variable Y, which is characterized by Rösler (1991). An even stronger convergence is true, where (absolute) moments converge to (absolute) moments of this limiting distribution (Wasserstein d_p convergence, for any p) (Rösler 1991). Thus:

$$\begin{aligned}\mathbb{E}[|I_S-\mathbb{E}[I_S]|^p] &\sim \mathbb{E}[|Y|^p]n^p,\\ \mathbb{E}[(I_S-\mathbb{E}[I_S])^p] &\sim \mathbb{E}[Y^p]n^p.\end{aligned} \tag{A12}$$

Furthermore, recalling that the expectation of I_S is equivalent to $2n\ln n$ and from rearranging (using continuous mapping) we note that:

$$\frac{I_S-\mathbb{E}[I_S]}{n} \overset{d}{\sim} Y \Rightarrow \frac{I_S}{\mathbb{E}[I_S]} \overset{p}{\sim} 1 \Rightarrow J^1 = \frac{n\log_2 n}{I_S} \overset{p}{\sim} \frac{1}{2\ln 2}. \tag{A13}$$

So, asymptotically, $J^1 \approx \frac{1}{2\ln 2} \approx 0.72$ (in probability, which we also see from simulations).

Now, we characterize the error term associated with the 2nd order Taylor approximation ($\mathbb{E}[R_k(I_S)]$ for $k=2$). Using (A9), the error can be written as $\mathbb{E}\left[\frac{(I_S-\mathbb{E}[I_S])^3}{z(I_S)^4}\right]$. Since $z(I_s) \in [\min\{\mathbb{E}[I_S], I_S\}, \max\{\mathbb{E}[I_S], I_S\}]$ and by continuous mapping $\frac{\min\{\mathbb{E}[I_S],I_S\}}{\mathbb{E}[I_S]} \overset{p}{\sim} 1$, $\frac{\max\{\mathbb{E}[I_S],I_S\}}{\mathbb{E}[I_S]} \overset{p}{\sim} 1$ (using (A13)), so $z(I_S)/\mathbb{E}[I_S] \overset{p}{\sim} 1$. This motivates (but does not yet show) the following asymptotic approximation to the error term:

$$\frac{\mathbb{E}\left[\frac{(I_S-\mathbb{E}[I_S])^3}{z(I_S)^4}\right]}{\mathbb{E}\left[\frac{(I_S-\mathbb{E}[I_S])^3}{\mathbb{E}[I_S]^4}\right]} \to 1$$

Showing the above is equivalent to showing:

$$\frac{\mathbb{E}\left[(I_S-\mathbb{E}[I_S])^3\left(\frac{\mathbb{E}[I_S]^4}{z(I_S)^4}-1\right)\right]}{\mathbb{E}[(I_S-\mathbb{E}[I_S])^3]} \to 0.$$

Using Cauchy-Schwarz:

$$\begin{aligned}\left|\frac{\mathbb{E}\left[(I_S-\mathbb{E}[I_S])^3\left(\frac{\mathbb{E}[I_S]^4}{z(I_S)^4}-1\right)\right]}{\mathbb{E}[(I_S-\mathbb{E}[I_S])^3]}\right| &\le \frac{\sqrt{\mathbb{E}\left[(I_S-\mathbb{E}[I_S])^6\right]\mathbb{E}\left[\left(\frac{\mathbb{E}[I_S]^4}{z(I_S)^4}-1\right)^2\right]}}{|\mathbb{E}[(I_S-\mathbb{E}[I_S])^3]|}\\ &\sim \frac{\sqrt{\mathbb{E}[Y^6]}}{|\mathbb{E}[Y^3]|}\sqrt{\mathbb{E}\left[\left(\frac{\mathbb{E}[I_S]^4}{z(I_S)^4}-1\right)^2\right]}.\end{aligned}$$

It then suffices to show $\mathbb{E}\left[\left(\frac{\mathbb{E}[I_S]^4}{z(I_S)^4}-1\right)^2\right]\to 0$. Define the set

$$A=\{\omega : I_S(\omega)\geq \mathbb{E}[I_S]/2\}.$$

and let A^c denote its complement. Noting that:

$$\mathbb{E}\left[\left(\frac{\mathbb{E}[I_S]^4}{z(I_S)^4}-1\right)^2\right]=\mathbb{E}\left[\left(\frac{\mathbb{E}[I_S]^4}{z(I_S)^4}-1\right)^2\mathbf{1}_A\right]+\mathbb{E}\left[\left(\frac{\mathbb{E}[I_S]^4}{z(I_S)^4}-1\right)^2\mathbf{1}_{A^c}\right]$$

it suffices to prove that each summand converges to 0. The strategy to control the first term is to show that on the event A, the expectation decays to 0 as $n\to\infty$. To control the second term, we show that the probability of A^c decays to 0 sufficiently quickly as $n\to\infty$.

On set A, $\frac{\mathbb{E}[I_S]}{z(I_S)}\leq 2$, so

$$\begin{aligned}\left|\frac{\mathbb{E}[I_S]^4}{z(I_S)^4}-1\right|&=\left|\frac{\mathbb{E}[I_S]}{z(I_S)}-1\right|\left|\left(\frac{\mathbb{E}[I_S]}{z(I_S)}\right)^3+\left(\frac{\mathbb{E}[I_S]}{z(I_S)}\right)^2+\frac{\mathbb{E}[I_S]}{z(I_S)}+1\right|\\&\leq 15\left|\frac{\mathbb{E}[I_S]}{z(I_S)}-1\right|=15\left|\frac{\mathbb{E}[I_S]-z(I_S)}{z(I_S)}\right|\\&\leq 15\left|\frac{\mathbb{E}[I_S]-I_S}{z(I_S)}\right|\leq 30\frac{|I_S-\mathbb{E}[I_S]|}{\mathbb{E}[I_S]}.\end{aligned}$$

Thus:

$$\mathbb{E}\left[\left(\frac{\mathbb{E}[I_S]^4}{z(I_S)^4}-1\right)^2\cdot\mathbf{1}_A\right]\leq\frac{900}{\mathbb{E}[I_S]^2}\mathbb{E}\left[|I_S-\mathbb{E}[I_S]|^2\right]=\mathcal{O}\left(\frac{1}{(\ln n)^2}\right)\to 0.$$

On the complement of set A (that is, on A^c), $I_S\leq\mathbb{E}[I_S]/2\leq\mathbb{E}[I_S]$ so $z(I_S)\leq\mathbb{E}[I_S]$. Moreover, due to the trivial bound $I_S\geq n$, we have $z(I_S)\geq n$. Hence

$$\left|\frac{\mathbb{E}[I_S]^4}{z(I_S)^4}-1\right|=\frac{\mathbb{E}[I_S]^4}{z(I_S)^4}-1\leq\frac{\mathbb{E}[I_S]^4}{z(I_S)^4}\leq\widetilde{C}(\ln n)^4$$

for some constant $\widetilde{C}$. Notice further that

$$\begin{aligned}\mathbb{P}\left(I_S<\frac{\mathbb{E}[I_S]}{2}\right)&\leq\mathbb{P}\left(|I_S-\mathbb{E}[I_S]|>\frac{\mathbb{E}[I_S]}{2}\right)=\mathbb{P}\left(|I_S-\mathbb{E}[I_S]|^9>\left(\frac{\mathbb{E}[I_S]}{2}\right)^9\right)\\&\leq\frac{2^9\mathbb{E}[|I_S-\mathbb{E}[I_S]|^9]}{\mathbb{E}[I_S]^9}=\mathcal{O}\left(\frac{1}{(\ln n)^9}\right).\end{aligned}$$

Hence

$$\mathbb{E}\left[\left(\frac{\mathbb{E}[I_S]^4}{z(I_S)^4}-1\right)^2\cdot\mathbf{1}_{A^c}\right]\le\widetilde{C}^2(\ln n)^8\mathbb{P}(A^c)$$
$$\le\frac{2^9\mathbb{E}[|I_S-\mathbb{E}[I_S]|^9]}{\mathbb{E}[I_S]^9}\widetilde{C}^2(\ln n)^8=\mathcal{O}\left(\frac{1}{\ln n}\right)\to 0.$$

Finally then

$$\mathbb{E}\left[\left(\frac{\mathbb{E}[I_S]^4}{z(I_S)^4}-1\right)^2\right]=\mathbb{E}\left[\left(\frac{\mathbb{E}[I_S]^4}{z(I_S)^4}-1\right)^2\mathbf{1}_A\right]+\mathbb{E}\left[\left(\frac{\mathbb{E}[I_S]^4}{z(I_S)^4}-1\right)^2\mathbf{1}_{A^c}\right]\to 0.$$

So, we can indeed say that

$$\mathbb{E}\left[\frac{(I_S-\mathbb{E}[I_S])^3}{z(I_S)^4}\right]\sim\mathbb{E}\left[\frac{(I_S-\mathbb{E}[I_S])^3}{\mathbb{E}[I_S]^4}\right]. \tag{A14}$$

Thus using Eqs. A14 and A12:

$$\mathbb{E}[n\log_2 n\cdot R_2(I_S)]\sim\frac{n\log_2 n}{(2n\ln n)^4}\mathbb{E}[Y^3]n^3=\frac{\mathbb{E}[Y^3]}{16\ln 2}\frac{1}{(\ln n)^3}.$$

Expressions for moments of Y are given, in a rather cumbersome recursive form, by Rösler (1991). In general, for a kth order Taylor approximation, following the same steps as above,

$$\mathbb{E}[(n\log_2 n)R_k(I_S)]\sim\frac{\mathbb{E}[Y^{k+1}]}{2^{k+2}\ln 2(\ln n)^{k+1}}.$$

Since $\mathbb{E}[Y^2]$ is simply the leading term coefficient of $\mathbb{V}[I_S]$, we verify in Figure 2 that the theoretical error for the first-order approximation is close to the empirical error.

Full m-ary generalized Yule trees

Although m-ary trees are less prevalent than bifurcating trees in the evolutionary literature, we note that the above procedure can still be easily generalized as follows.

Consider a generalized Yule process in which a leaf is selected uniformly at random and replaced by a star subtree of outdegree m. For general m-ary trees generated under this generalized Yule process, we have:

$$\mathbb{E}[I_S]=(1+\theta)n\left(\gamma(i+\theta+1)-\gamma(\theta+1)\right)$$
$$\mathbb{V}[I_S]=n^2\left((2+\theta)\frac{i}{i+\theta}-(1+\theta)^2(\gamma'(\theta+1)-\gamma'(i+\theta+1))-\frac{1}{i+\theta}(\gamma(i+\theta+1)-\gamma(\theta+1))\right)$$

where $\theta = \frac{1}{m-1}$, i is the number of internal nodes, which satisfies $n = (m-1)i + 1$ and hence $i = (n-1)/(m-1)$, and γ is the digamma function (Halton 1989). Thus, $\mathbb{E}[I_S] \sim (1+\theta) \cdot n \ln n$ and $\mathbb{V}[I_S] \sim C_2 \cdot n^2$. Therefore:

$$\mathbb{P}\left(\left|\frac{I_S}{\mathbb{E}[I_S]} - 1\right| > \varepsilon\right) = \mathbb{P}(|I_S - \mathbb{E}[I_S]| > \mathbb{E}[I_S]\varepsilon) \leq \frac{\mathbb{V}[I_S]}{(\mathbb{E}[I_S]\varepsilon)^2} \to 0.$$

So $\frac{I_S}{\mathbb{E}[I_S]} \overset{p}{\sim} 1$. Using the leafy tree identity (Proposition 1) and continuous mapping, this implies that $J^1 \overset{p}{\sim} \frac{1}{(1+\theta)\ln m}$. Thus, we may apply similar analysis to above (Section A.2.1) to show that a kth order Taylor approximation's error would decay at rate $1/(\ln n)^{(k+1)}$.

A.2.2 Uniform

Under this model, each tree of n leaves is as likely as any other. It is important to note that the uniform model selects higher Sackin-index trees than the Yule model.

Let $X_n = \frac{I_{S,n}}{n^{3/2}}$. Under the uniform model, $\lim_{n\to\infty} \mathbb{E}[X_n^r] = \mathbb{E}[A^r]$ (Takács 1991), where A is the Airy distribution and $r \in \mathbb{Z}^+$. Such moments are quite simple to obtain:

$$\mathbb{E}[A^r] = \sqrt{8}^r n^{3r/2} M_r$$

where

$$M_r = K_r \cdot \frac{4\sqrt{\pi} r!}{\Gamma\left(\frac{3r-1}{2}\right) 2^{r/2}}, K_r = \frac{3r-4}{4} K_{r-1} + \sum_{j=1}^{r-1} K_j K_{r-j}$$

and $K_0 = -\frac{1}{2}$ (Takács 1991).

We have exact expressions for the mean and variance of I_S under the uniform model, but the above can be used to generate kth-order Taylor approximations of $\mathbb{E}[J^1]$ for $k \geq 3$ using asymptotic approximations of moments if desired. Further, note that $\mathbb{E}[A^{-1}] = \frac{\sqrt{\pi}}{4}\left(3 - 16\frac{\pi^3\sqrt{3}}{\Gamma(1/3)^6}\right)$ (Flajolet and Louchard 2001). Thus, conjecturing on the convergence of the inverse moment $\mathbb{E}[X_n^{-1}] \to \mathbb{E}[A^{-1}]$, we obtain the following large n approximation:

$$\mathbb{E}[J^1] \sim \mathbb{E}[A^{-1}] n^{-1/2} \log_2 n,$$

which is shown in Figure 2.

A.3 Derivation of Equation 12

For brevity we present the derivation in the case of equally sized leaves. The derivation for general $p > 0$ is similar.

$$\begin{aligned} J_B^1(n,k,1) &= \frac{1}{\sum_{l=k}^{n} l} \sum_{i\in\tilde{V}} S_i^* \sum_{j\in C(i)} W_{ij}^1 \\ &= \frac{-2}{(n+k)(n-k+1)} \sum_{i\in\tilde{V}} S_i^* \sum_{j\in C(i)} \frac{S_j}{S_i^*} \log_{d^+(i)} \frac{S_j}{S_i^*} \end{aligned}$$

$$
\begin{aligned}
&= \frac{-2}{(n+k)(n-k+1)} \left(\sum_{\substack{i \in \tilde{V} \\ d^+(i)=2}} S_i^* \sum_{j \in C(i)} \frac{S_j}{S_i^*} \log_2 \frac{S_j}{S_i^*} + k \cdot k \cdot \frac{1}{k} \log_k \frac{1}{k} \right) \\
&= \frac{-2}{(n+k)(n-k+1)} \left(\sum_{\substack{i \in \tilde{V} \\ d^+(i)=2}} S_i \left(\frac{S_i - 1}{S_i} \log_2 \frac{S_i - 1}{S_i} + \frac{1}{S_i} \log_2 \frac{1}{S_i} \right) - k \right) \\
&= \frac{2}{(n+k)(n-k+1)} \left(\sum_{i=k+1}^{n} i \left(\frac{i-1}{i} \log_2 \frac{i}{i-1} + \frac{1}{i} \log_2 i \right) + k \right) \\
&= \frac{2}{(n+k)(n-k+1)} \left(\sum_{i=k+1}^{n} \left((i-1) \log_2 \frac{i}{i-1} + \log_2 i \right) + k \right) \\
&= \frac{2}{(n+k)(n-k+1)} \left(\log_2 \frac{n^n k!}{k^k n!} + \log_2 \frac{n!}{k!} + k \right) \\
&= \frac{2}{(n+k)(n-k+1)} \left(\log_2 \frac{n^n}{k^k} + k \right) \\
&= \frac{2(n \log_2 n - k \log_2 k + k)}{(n+k)(n-k+1)}.
\end{aligned}
$$

A.4 Expanded explanation as to why caterpillars are not always the least balanced broom trees

Let T be the leafy caterpillar tree with five leaves, each of size 1, and number its four internal nodes from the deepest up to the root, starting at 1. The balance score of node i is then

$$
\begin{aligned}
W_i &= -\frac{i}{i+1} \log_2 \frac{i}{i+1} - \frac{1}{i+1} \log_2 \frac{1}{i+1} \\
&= \log_2(i+1) - \frac{i}{i+1} \log_2 i.
\end{aligned}
$$

Thus $W_1 = 1$ (because node 1 is the root of the two-leaf broom head), $W_2 \approx 0.92, W_3 \approx 0.81$, and $W_4 \approx 0.72$. The weight assigned to node i is $S_i = i + 1$. Hence

$$
J^1(T) = \frac{5W_4 + 4W_3 + 3W_2 + 2W_1}{5 + 4 + 3 + 2} \approx 0.8293.
$$

This result can be checked using the general formula for caterpillar trees with $n = 5$:

$$
J^1(T) = \frac{2n \log_2 n}{(n-1)(n+2)} \approx 0.8293.
$$

Now suppose we modify T by removing the second lowest internal node (node 2) and reattaching its child leaf to the broom head, to create tree T'. Since node 2 was the second most balanced node in T, its removal decreases J^1. But the removal of node 2 also decreases the normalizing factor of J^1 (here equal to Sackin's index) from 14 to 12, which increases J^1. Nodes 3 and 4 have the same weights and balance scores as before but the weight of node 1 (the root of the broom head, which remains maximally balanced) increases from 2 to 3. Hence

$$J^1(T') = \frac{5W_4 + 4W_3 + 3W_1}{5 + 4 + 3} \approx 0.8212.$$

In summary, we have two factors that increase J^1 (decreasing the denominator and assigning more weight to $W_1 = 1$ in the numerator) and one that decreases J^1 (removing W_2 from the numerator). The latter effect dominates in this example and in all other cases where the leaf count is greater than 4 (Proposition 4).

More generally, let

$$A = \frac{1}{q_n}\left(nW_{n-1} + \cdots + 4W_3 + 3W_2 + 2W_1\right)$$

denote the J^1 value of the caterpillar tree with $n > 2$ leaves (so $n-1$ internal nodes), where $q_n = n + \ldots 4 + 3 + 2$, and let

$$B = \frac{1}{q_n - 2}\left(nW_{n-1} + \cdots + 4W_3 + 3W_1\right)$$

denote the J^1 value of the broom tree created by removing the second lowest internal node and reattaching its child leaf to the broom head. Let $C = (3W_2 - W_1)/2$. Then

$$B - A = \frac{2(A - C)}{q_n - 2},$$

where $q_n - 2 > 0$.

Thus, if $A < C$ (that is, if the balance index of the caterpillar tree is less than the fixed quantity C) then $B < A$ (that is, the non-caterpillar broom tree is less balanced than the caterpillar). But A decreases monotonically with n (the caterpillar becomes less balanced as it grows). Therefore, once A becomes smaller than C (when $n = 4$), it remains smaller for all larger values of n.

A.5 Proof of Proposition 6

A.5.1 Minimum

We first prove the formulas for the equivalent of $\min_{2 \le k \le n} J^1_B(n, k, p)$ as $n \to +\infty$. Recall that, letting $r = k/n$:

$$J^1_B(n, k, p) = 2\frac{(1-r)\log_2 n + (1 - r + pr)\log_2(1 - r + pr) - pr\log_2 pr + pr}{(1 + (2p-1)r)(n(1-r) + 1)}. \quad \text{(A15)}$$

Consider an arbitrary sequence (k_n) with $2 \leq k_n \leq n$. Let $r_n = k_n/n$. Assume first that $(1-r_n)\ln n \to +\infty$. Then $(1-r_n)n \to +\infty$, and it follows from (A15) that:

$$J_B^1(n, k_n, p) \sim \frac{2}{1+(2p-1)r_n} \times \frac{\log_2 n}{n}. \tag{A16}$$

In particular, if $r_n \to r < 1$ (which implies that $(1-r_n)\ln n \to +\infty$), then:

$$J_B^1(n, k_n, p) \sim \frac{2}{1+(2p-1)r} \times \frac{\log_2 n}{n}. \tag{A17}$$

From now on, let (k_n) be an optimal sequence and $r_n = k_n/n$. Consider three cases:
Case 1: $p > 1/2$**.** It follows from (A17) that $r_n \to 1$. Therefore, due to (A15),

$$J_B^1(n, k_n, p) \sim \frac{(1-r_n)\log_2 n + p}{pn(1-r_n+1/n)} = \frac{1}{pn}\left[\log_2 n + \frac{p - \frac{\log_2 n}{n}}{1-r_n+\frac{1}{n}}\right] \geq \frac{\log_2 n}{pn}, \tag{A18}$$

where the inequality holds for n large enough. Thus the smallest rate we can hope for is $\frac{\log_2 n}{pn}$. By (A16), this is (only) achieved by any sequence (k_n) such that $r_n \to 1$ and $(1-r_n)\ln n \to +\infty$, that is, that converges to 1 sufficiently slowly. We conclude that

$$\min_{2\leq k\leq n} J_B^1(n, k, p) \sim \frac{\log_2 n}{pn}.$$

Case 2: $p < 1/2$. Assume that r_n is bounded away from 1. Then if follows from (A16) that

$$\min_{2\leq k\leq n} J_B^1(n, k, p) \sim \frac{2\log_2 n}{n} \tag{A19}$$

and that this is achieved if and only if $r_n \to 0$. But r_n is indeed bounded away from 1. Otherwise, a subsequence of (r_n) would converge to 1. The proof for the case $p > 1/2$ shows that, along this subsequence, the best rate that could be obtained would be $\frac{\log_2 n}{pn}$. Since we now assume $p < 1/2$, this is larger than $\frac{2\log_2 n}{n}$, hence suboptimal. This completes the proof of (A19). The proof also shows that $r_n \to 0$.

Case 3: $p = 1/2$. Due to (A16), any sequence bounded away from 1 leads to the rate $\frac{2\log_2 n}{n}$. Moreover, previous arguments show that the best one can get from a sequence or subsequence going to 1 is the same rate. Thus (A19) holds as well, and up to second order terms, all sequences (r_n) except those converging very quickly to 1 lead to the asymptotic behaviour. Note that (A19) also follows from Proposition 7.

A.5.2 Minimizers

Case 1: $p > \frac{1}{2}$

Consider the sequence of arg minimums when treating r as a variable taking values on $[\frac{2}{n}, 1]$ (in which case, it is labeled x)

$$x_n^* = \arg\min_{x\in[\frac{2}{n},1]} J_B^1(n, xn, p). \tag{A20}$$

Consider the partial derivative of $f_n(x,p) = J(n, nx, p)$ with respect to x:

$$\frac{\partial f_n(x,p)}{\partial x} = 2\frac{A_p(x)n + B_p \ln n + C_p(x) + D_p(x)n \ln n}{(x(2p-1)+1)^2(n(1-x)+1)^2 \ln 2}, \tag{A21}$$

where

$$A_p(x) = -1 + 2x(1-p) + (2p-1)x^2 + p\ln 2 - px^2 \ln 2 + 2p^2x^2 \ln 2 - p\ln(px) \\ -p(2p-1)x^2 \ln(px) + ((x-1)^2 + 2p^2x^2 + p(x-1)(1-3x))\ln(1+x(p-1)), \tag{A22}$$

$$B_p = -2p, \tag{A23}$$

$$C_p(x) = -1 + x - 2px + p\ln 2 - p\ln(px) - p\ln(1+x(p-1)), \tag{A24}$$

$$D_p(x) = (1-2p)(x-1)^2. \tag{A25}$$

First, we prove that the discrete and continuous minimizers are close $|x_n^* - r_n^*| \leq \frac{1}{n}$. Using similar arguments as in Appendix A.5.1, $x_n^* \to 1$. Let g_n denote the numerator of A21. Since $A_p'(1) > 0$ and $D_p'(x) > 0$ on $[0,1)$ and $C_p'(x)$ is bounded on a neighborhood of 1, it follows that there is an $\varepsilon > 0$ such that on $x \in [1-\varepsilon, 1]$, $A_p'(x) > a > 0$ and $C_p'(x) > b$. So, we can choose N, depending on only on a and b, such that $\forall n \geq N$ on $x \in [1-\varepsilon, 1]$:

$$g_n'(x) = A_p'(x)n + C_p'(x) + D_p'(x)n\ln n \geq A_p'(x)n + C_p'(x) \geq an + b > 0$$

So, $g_n'(x) > 0$ on $[1-\varepsilon, 1]$ for all n sufficiently large. Eventually, for large enough n, $r_n^*, x_n^* \in [1-\varepsilon, 1]$. g_n is thus initially negative, then zero at $x = x_n^*$, then positive. So, $x \to f_n(x,p)$ strictly decreases until $x = x_n^*$, then strictly increases (on $[1-\varepsilon, 1]$). For $n \geq N$ and such that $\frac{3}{n} < \varepsilon$, it follows that r_n^* must be the multiple of $1/n$ that comes immediately after or before x_n^* (or both). So, $|x_n^* - r_n^*| < \frac{1}{n}$.

Furthermore,

$$\frac{\partial f_n(x,p)}{\partial x}(x_n^*) = 0 \iff 0 = A_p(x_n^*)n + B_p \ln n + C_p(x_n^*) + D_p(x_n^*)n\ln n. \tag{A26}$$

Observe that $A_p(1) = 2p^2 \ln 2 \neq 0$, hence in the limit with respect to n:

$$\begin{aligned} D_p(x_n^*)n\ln n &\sim -A_p(x_n^*)n, \\ D_p(x_n^*)\ln n &\sim -(2p^2 \ln 2), \\ (2p-1)(1-x_n^*)^2 &\sim \frac{2p^2}{\log_2 n}, \\ 1 - x_n^* &\sim \frac{p\sqrt{2}}{\sqrt{(2p-1)\log_2 n}}. \end{aligned} \tag{A27}$$

Using $|x_n^* - r_n^*| \leq \frac{1}{n}$, the above result, and the Squeeze Theorem, the proof is complete.

Case 2: $p < \frac{1}{2}$

For all $x \in [0, \frac{1}{2}]$, $nD_p(x) - B_p \geq \frac{n(1-2p)}{4} - 2p > 0$ for n sufficiently large. Moreover, $A_p(x), C_p(x) \to \infty$ as $x \to 0^+$. Hence by A21, there is a $\varepsilon > 0$ such that for all sufficiently large n, $x \to f_n(x,p)$ is strictly increasing on $(0, \varepsilon] \supseteq [\frac{2}{n}, \varepsilon]$. From Appendix A.5.1, $r_n^* \to 0$ for $p < \frac{1}{2}$. Eventually then, for all n large enough, $r_n^* \in [\frac{2}{n}, \varepsilon]$. But because $x \to f_n(x,p)$ is strictly increasing within this interval, $r_n^* = \frac{2}{n}$, or equivalently $k_n^* = 2$, for all sufficiently large n.

Case 3: $p = \frac{1}{2}$

This follows from Proposition 7

A.6 Proof of Proposition 7

Proof

$$\frac{\partial J_B^1}{\partial k}\left(n, k, \frac{1}{2}\right) = \frac{-(n+1)\ln k + (n-1)\ln(2n-k) - 2(n+1-k) + (n+3)\ln 2}{n(n+1-k)^2 \ln 2}$$
$$= \frac{n \ln\left(\frac{2n}{k} - 1\right) - \ln(k(2n-k)) - 2(n+1-k) + (n+3)\ln 2}{n(n+1-k)^2 \ln 2}.$$

We then note that, for all $2 \leq k \leq n$,

$$n \ln\left(\frac{2n}{k} - 1\right) = 2\left(n - k + \frac{(n-k)^3}{3n^2} + \frac{(n-k)^5}{5n^4} + \dots\right) \geq 2(n-k)$$

and $\ln(k(2n-k)) \leq 2\ln n$. Hence

$$\frac{\partial J_B^1}{\partial k}\left(n, k, \frac{1}{2}\right) \geq \frac{(n+3)\ln 2 - 2(\ln n + 1)}{n(n+1-k)^2 \ln 2},$$

which is positive for all $n \geq 4$. Therefore $J_B^1(n, k, \frac{1}{2})$ increases with k, which implies it is minimal when $k = 2$. □

A.7 Proof of Proposition 8

Proof We first reparameterize J_B^1 as

$$J_B^1(n, rn, p) = \frac{2}{n} \times \frac{(1-r)\log_2 n + (1-r+pr)\log_2(1-r+pr) - pr\log_2 pr + pr}{(1+(2p-1)r)(1-r+1/n)}.$$

As $p \to \infty$

Let n be fixed throughout. Firstly note that for fixed n and k, as $p \to \infty$,

$$pr\log_2(1-r+pr) - pr\log_2(pr) = \frac{pr}{\ln 2}\ln\left(1 + \frac{1-r}{pr}\right) \sim \frac{pr}{\ln 2} \times \frac{1-r}{pr} = \frac{1-r}{\ln 2}$$

Thus, the leading term is $2pr$ in the numerator and $2pr(n - nr + 1)$ in the denominator. Therefore, $(p \to J_B^1(n,k,p)) \sim \frac{1}{n-nr+1}$. For fixed n and large enough p, $r^* = \frac{2}{n}$ (a constant with respect to p). This may be seen by using (A21) and noticing that the leading term in p in the numerator is $4p^2x^2\ln(1 + x(p-1))n$ for any $x \in \left[\frac{2}{n}, 1\right]$. It follows that there exists P such that, for any $p > P$, f_n is increasing in x over this interval, hence so is J_B^1 in terms of $r \in \{\frac{2}{n}, \frac{3}{n}, \dots, \frac{n}{n}\}$. Therefore, $J_B^1(n, k^*, p) \sim \frac{1}{n-1}$.

As $p \to 0$

Let n be fixed. We first show that for p small enough, we have $k^* = n-1$. Notice that:

$$\lim_{p \to 0^+} J_B^1(n,k,p) = \frac{2\log_2(n-k)}{n-k+1} \equiv J_B^1(n,k,0)$$

for $k \in \{2,\dots,n-1\}$ and for $k=n$, $\lim_{p\to 0^+} J_B^1(n,k,p) = 1$. We find the critical points of $J_B^1(n,k,0)$ at fixed n, which becomes

$$2\left[\frac{\frac{1}{(n-k)\ln 2}}{n-k+1}\cdot(-1) + \frac{\log_2(n-k)}{(n-k+1)^2}\right] = 0$$

$$\Leftrightarrow n-k+1 = (n-k)\ln(n-k) \Leftrightarrow 1 = (\ln(n-k)-1)e^{\ln(n-k)}$$

$$\Leftrightarrow k = n - e^{W_0(e^{-1})+1}$$

where W_0 is the real, increasing branch of the Lambert W Function. This is on the interior of $[2, n-1]$ for $n \geq 6$, is a unique critical point, and is the maximum of $J_B^1(n,k,0)$ for fixed n. Thus, the global minimum must be on 2 or $n-1$. By direct computation $0 = J_B^1(n,n-1,0) < J_B^1(n,2,0)$, proving $k^* = n-1$.

So,

$$J_B^1(n,k^*,p) = \log_2 n + (1+p(n-1))\log_2(\frac{1}{n} + p\frac{n-1}{n}) - p(n-1)\log_2(p\frac{n-1}{n}) + p(n-1).$$

As $x \to 0^+$, terms of the forms $t(x) = x\ln(1+x), t(x) = \ln(1+x), t(x) = x$ decay much faster to 0 compared to $x \ln x$ (meaning $\lim_{x\to 0^+} \frac{t(x)}{x\ln x} = 0$), so asymptotically:

$$J_B^1(n,k^*,p) \sim p(1-n)\log_2(p).$$

□

A.8 Proof of Proposition 9

R_3-R_4 boundary

The approximate condition to be on the boundary is $J_B^1(n,2,p) = J_B^1(n,n-1,p)$ (approximate, as n is assumed to be continuous), which is equivalent to

$$\frac{2[(2p+n-2)\log_2(2p+n-2) - 2p\log_2(p)]}{(4p+n-2)(n-1)} = \frac{[(n-1)p + ((n-1)p+1)\log_2((n-1)p+1) - (n-1)p\log_2(n-1)p]}{2(n-1)p+1}.$$

Note that one key fact about the boundary is that as $n \to \infty$, $p \to 0^+$. Notice that we have the following possible options for the asymptotic behavior of (n,p):

1. As $n \to +\infty$, p is bounded below
2. As $p \to 0^+$, n is bounded above
3. As $n \to +\infty$, $p \to 0^+$

The first and second option are precluded by Proposition 6 and Proposition 8. Now then:

$$\begin{aligned}
&\frac{2\log_2 n}{n} = \frac{[np + (np+1)\log_2(np+1) - np\log_2 np]}{2np+1} \\
\Leftrightarrow\quad &\frac{2(2np+1)\log_2 n}{n} - np - (np+1)\log_2(np+1) + np\log_2 np = 0.
\end{aligned}$$

Since $np \sim np + p\log_2 n$, we can further simplify:

$$-\frac{2\log_2 n}{n} + np + (np+1)\log_2(np+1) - np\log_2 np = 0,$$

and then

$$np\log_2(np+1) - np\log_2 np = -\log_2(np+1) + \frac{2\log_2 n}{n} - np. \tag{A28}$$

Notice the left-hand side is always positive, meaning

$$\begin{aligned}
&-\log_2(np+1) + \frac{2\log_2 n}{n} - np > 0 \\
\Leftrightarrow\quad & n^{2/n} > (np+1)e^{np\ln 2} \\
\Leftrightarrow\quad & n^{2/n}\ln 4 > (np\ln 2 + \ln 2)e^{np\ln 2 + \ln 2} \\
\Leftrightarrow\quad & W_0(n^{2/n}\ln 4) > np\ln 2 + \ln 2 \\
\Leftrightarrow\quad & \frac{W_0(e^{2\ln n/n}\ln 4) - \ln 2}{n\ln 2} > p,
\end{aligned}$$

where W_0 denotes the real, increasing branch of the Lambert W function.

Note that as $n \to \infty$, $W_0(e^{2\ln n/n}\ln 4) \to W_0(\ln 4)$. Additionally, a point on the graph of $xe^x = y$ is $(\ln 2, \ln 4)$, meaning $W_0(\ln 4) = \ln 2$. Finally, $W_0'(x) = \frac{W_0(x)}{x(W_0(x)+1)}$, so $W_0'(\ln 4) = \frac{\ln 2}{\ln 4(\ln 2+1)} = \frac{1}{2(\ln 2+1)}$. Using the first order approximation of $W_0(x)$ centered at $\ln 4$ and e^x centered at 0 ($\ln 2 + \frac{1}{2(\ln 2+1)}(x - \ln 4)$ and $1+x$, respectively), we obtain the asymptotic result:

$$\begin{aligned}
&\frac{\ln 2 + \frac{1}{2(\ln 2+1)}((1 + 2\ln n/n)\ln 4 - \ln 4) - \ln 2}{n\ln 2} > p \\
\Leftrightarrow\quad &\frac{\frac{1}{(\ln 2+1)}(2\ln n/n)}{n} > p \quad\Leftrightarrow\quad \frac{2\ln n}{(\ln 2+1)n^2} > p.
\end{aligned}$$

Note further that asymptotically $\frac{2\ln n}{(\ln 2+1)n} > np$, so namely $np \to 0$. Furthermore, in the limit $np \to 0$, $np\log_2(np)$ goes to 0 much slower than any other np term in

A28, so we reduce A28 to:

$$np \log_2 np = -\frac{2 \log_2 n}{n} \quad \Leftrightarrow \quad (np)^{np} = n^{-\frac{2}{n}}.$$

Notice the above becomes:

$$\ln(np)e^{\ln(np)} = -\frac{2}{n}\ln n \quad \Leftrightarrow \quad \ln(np) = W_{-1}(-\frac{2}{n}\ln n) \quad \Leftrightarrow \quad p = \frac{-\frac{2}{n}\ln n}{nW_{-1}(-\frac{2}{n}\ln n)},$$

where $W_{-1}(x)$ is the decreasing, real branch of the Lambert W function. As $x \to 0^-$, $W_{-1}(x) \to -\infty$ and $W_{-1}(x) \sim \ln(-x)$, so asymptotically we obtain

$$p \overset{n\to\infty}{\sim} \frac{-\frac{2}{n}\ln n}{n\ln(\frac{2}{n}\ln n)} = \frac{-\frac{2}{n}\ln n}{n(\ln 2 - \ln n + \ln\ln n)} \overset{n\to\infty}{\sim} \frac{2}{n^2}.$$

R_1-R_2 boundary

A similar analysis to that presented above as $p \to \infty$ and $n \to \infty$ shows that the R_1-R_2 boundary approaches $p = n^2 \log_2 n/3$.

A.9 Proof of Proposition 11

Proof Since J_B^1 is a continuous function of p, $\min_{2\le k\le n} J_B^1(n,k,p)$ is also a continuous function of p, so it suffices to prove the result separately for each region of n-p space.

Regions R_1 and R_3

By Proposition 10, the result holds in the regions R_1 and R_3 where $k^* = 2$.

Region R_2

If $p > \frac{1}{2}$ then $\theta(k,p) < 4/k + k/2 < k$ for all $k \ge 3$. We then have $n \ge k > \theta(k,p)$ for all $n \ge 3$, $k \in \{3,\dots,n\}$ and $p > \frac{1}{2}$, which, by Proposition 7, includes the region R_2. Therefore, by Proposition 10, $J_B^1(n,k,p)$ is a strictly decreasing function of p for all $(n,p) \in R_2$ and all $k \in \{2,\dots,n-1\}$. It follows that in R_2, for any $\epsilon > 0$,

$$\begin{aligned}
\min_{2\le k\le n} J_B^1(n,k,p+\epsilon) &= \min_{3\le k\le n-2} J_B^1(n,k,p+\epsilon) \\
&= \min\{J_B^1(3,k,p+\epsilon),\dots,J_B^1(n,n-2,p+\epsilon)\} \\
&< \min\{J_B^1(3,k,p),\dots,J_B^1(n,n-2,p)\} \\
&= \min_{3\le k\le n-2} J_B^1(n,k,p) \\
&= \min_{2\le k\le n-1} J_B^1(n,k,p).
\end{aligned}$$

Hence $\min_{2\le k\le n} J_B^1(n,k,p)$ is a strictly decreasing function of p for all $(n,p) \in R_2$. Lastly, we have $\theta(2,p) = 1/p + 2(1-p) \le 3$ for all $p \ge \frac{1}{2}$, which implies $n > \theta(2,p)$ for all $(n,p) \in R_2$, as required.

Region R_4

First we note that

$$n < \theta(n-1,p) \Leftrightarrow p < \frac{1}{n-1} \Leftrightarrow n < \frac{p+1}{p}. \tag{A29}$$

Since $J_B^1(n, n-1, 1/(n-1)) = 1$, we cannot have $k^* = n-1$ along the curve $p = 1/(n-1)$, so the curve must lie entirely above R_4. Therefore $n < \theta(n-1,p)$ by Equation A29, and Proposition 10 then implies that $J_B^1(n, n-1, p)$ is a strictly increasing function of p for all $(n,p) \in R_4$. By Equation A29, $n < \theta(n-1,p)$ also implies

$$n < \frac{p+1}{p} < \frac{1}{p} + 2(1-p) = \theta(2,p)$$

for all $p < 1$, which, by Proposition 7, includes all p values in R_4. Hence, for all $(n,p) \in R_4$, we have shown first that $\min_{2 \le k \le n} J_B^1(n,k,p)$ is a strictly increasing function of p and second that $n < \theta(2,p)$, which proves the result for R_4. □

A.10 Proof of Corollary 11.1

Proof In the proof of Proposition 11, we showed that $n > \theta(2,p)$ for all $n > 3$ and $p > \frac{1}{2}$, which includes all of R_1 and R_2. We also showed that $n < \theta(2,p)$ for all $(n,p) \in R_4$. Therefore $n = \theta(2,p)$ must hold only in R_3, where $k^* = 2$. Hence when $\min_{2 \le k \le n} J_B^1(n,k,p)$ is maximal, $k^* = 2$ and $n = \theta(2,p)$, which has the unique positive solution

$$p = \frac{1}{4}\left(\sqrt{n^2 - 4n + 12} - n + 2\right).$$

Substituting this p value into $J_B^1(n,2,p)$ gives the required result. □

n	$\mathbb{H}_Y(I_S)$	$\mathbb{E}_Y(I_S)$	$\mathbb{E}_Y(J^1) = \frac{n \log_2 n}{\mathbb{H}_Y(I_S)}$	$\mathbb{H}_Y(J^1) = \frac{n \log_2 n}{\mathbb{E}_Y(I_S)}$	$\mathcal{J}(n) = \mathbb{E}_Y(J^1) - \mathbb{H}_Y(J^1)$
2	2	2	1	1	0
3	5	5	0.9509775	0.9509775	0
4	$\frac{216}{25}$	$\frac{26}{3}$	$0.\overline{925}$	$0.\overline{923076}$	$0.\overline{002849}$
5	$\frac{728}{57}$	$\frac{77}{6}$	0.9089966	0.9046473	0.0043493
6	$\frac{1162800}{67217}$	$\frac{87}{5}$	0.8965605	0.8913664	0.0051941
7	$\frac{199806750}{9017743}$	$\frac{223}{10}$	0.8869172	0.8812325	0.0056847
8	$\frac{14827566600}{543154091}$	$\frac{962}{35}$	0.8791529	0.8731809	0.0059720
9	$\frac{3276378490018233600}{100225924446507833}$	$\frac{4609}{140}$	0.8727252	0.8665883	0.0061369
10	$\frac{247828225931780785200}{6470294678155594501}$	$\frac{4861}{126}$	0.8672884	0.8610634	0.0062249
11	$\frac{4355154127379809158517035000}{98723552636267773621908727}$	$\frac{55991}{1260}$	0.8626104	0.8563470	0.0062634

Table A1 Harmonic and arithmetic means of I_S and J^1 under the Yule model, and corresponding values of the Jensen gap $\mathcal{J}(n)$. Non-repeating decimal numbers are rounded to seven decimal places. $H_Y(I_S)$ values are given as exact rational numbers to aid the search for a better general approximation or an exact formula.

n	$\mathbb{H}_U(I_S)$	$\mathbb{E}_U(I_S)$	$\mathbb{E}_U(J^1) = \frac{n \log_2 n}{\mathbb{H}_U(I_S)}$	$\mathbb{H}_U(J^1) = \frac{n \log_2 n}{\mathbb{E}_U(I_S)}$	$\mathcal{J}(n) = \mathbb{E}_U(J^1) - \mathbb{H}_U(J^1)$
2	2	2	1	1	0
3	5	5	0.9509775	0.9509775	0
4	$\frac{360}{41}$	$\frac{44}{5}$	$0.9\overline{1}$	$0.\overline{90}$	$0.0\overline{02}$
5	$\frac{3822}{289}$	$\frac{93}{7}$	0.8778614	0.8738439	0.0040174
6	$\frac{4883760}{267509}$	$\frac{386}{21}$	0.8495512	0.8437960	0.0057552
7	$\frac{2051349300}{86119541}$	$\frac{793}{33}$	0.8250067	0.8177793	0.0072274
8	$\frac{11813334132600}{395454217009}$	$\frac{12952}{429}$	0.8034058	0.7949351	0.0084707
9	$\frac{2440219396211496900}{67072134682154831}$	$\frac{26333}{715}$	0.7841601	0.7746351	0.0095250
10	$\frac{3265803434049000144300}{75388088210344282097}$	$\frac{106762}{2431}$	0.7668367	0.7564121	0.0104246

Table A2 Harmonic and arithmetic means of I_S and J^1 under the uniform model, and corresponding values of the Jensen gap $\mathcal{J}(n)$. Non-repeating decimal numbers are rounded to seven decimal places. $H_U(I_S)$ values are given as exact rational numbers to aid the search for a better general approximation or an exact formula.
